# Solvothermal Fabrication of Mesoporous Pd Nano-Corals at Mild Temperature for Alkaline Hydrogen Evolution Reaction

**DOI:** 10.3390/nano14100876

**Published:** 2024-05-17

**Authors:** Ming Zhao, Koh-ichi Maruyama, Satoshi Tanaka

**Affiliations:** 1 Department of Materials and Biology, National Institute of Technology, Akita College, 1-1 Iijimabunkyocho, Akita 011-8511, Akita, Japan; maruko@akita-nct.ac.jp; 2 Department of Materials Science and Technology, Nagaoka University of Technology, 1603-1 Kamitomioka, Nagaoka 252-1123, Niigata, Japan

**Keywords:** solvothermal synthesis, palladium, porous materials, hydrogen evolution

## Abstract

Porous metallic nanomaterials exhibit interesting physical and chemical properties, and are widely used in various fields. Traditional fabrication techniques are limited to metallurgy, sintering, electrodeposition, etc., which limit the control of pore size and distribution, and make it difficult to achieve materials with high surface areas. On the other hand, the chemical preparation of metallic nanoparticles is usually carried out with strong reducing agents or at high temperature, resulting in the formation of dispersed particles which cannot evolve into porous metal. In this study, we reported the simple fabrication of coral-like mesoporous Pd nanomaterial (Pd NC) with a ligament size of 4.1 nm. The fabrication was carried out by simple solvothermal reduction at a mild temperature of 135 °C, without using any templates. The control experiments suggested that tetrabutylammonium bromide (TBAB) played a critical role in the Pd(II) reduction into Pd nanoclusters and their subsequent aggregation to form Pd NC, and another key point for the formation of Pd NC is not to use a strong reducing agent. In alkaline water electrolysis, the Pd NC outperforms the monodisperse Pd NPs and the state-of-the-art Pt (under large potentials) for H_2_ evolution reaction, probably due to its mesoporous structure and large surface area. This work reports a simple and novel method for producing porous metallic nanomaterials with a high utilization efficiency of metal atoms, and it is expected to contribute to the practical preparation of porous metallic nanomaterials by solvothermal reductions.

## 1. Introduction

Palladium-based nanomaterials (PBMs) have been extensively studied as a type of hydrogenation catalyst and hydrogen storage material that shows strong metal hydride formation ability [1,2,3,4,5,6]. It has been reported that good hydrotreating catalysts could be logical candidates when initially screening electrode materials for hydrogen evolution reaction (HER) [7]. The calculated free energies of hydrogen adsorption (△G_H*_) on pure metals indicate that the value for Pd is close to that for Pt at high exchange current density [7,8,9,10]. Therefore, PBMs could become one of the most efficient types of HER catalysts. PBMs have been reported to exhibit high HER activity in acid electrolyte during very recent years [10]. Unfortunately, HER in alkaline medium, which dominates the commercial water electrolysis technology market, is much more sluggish, with the activity commonly two to three orders of magnitude lower than that in acids [11,12].

Porous materials, including coral-like structural nanomaterials, possess good conductivity due to their skeleton structure and high surface area, and can efficiently diffuse reactants (liquid and gas) due to their hollow structure. Furthermore, they show structural robustness, ascribed to their three-dimensional bicontinuous porous structure. These materials are considered to be ideal (active and durable) electrocatalysts for the electrolysis of H_2_O to H_2_, for example [10,13,14,15,16]. A conventional method for producing mesoporous metal without a template is to selectively elute a specific element from an alloy by dealloying or galvanic replacement. It requires sacrifice of a component and the use of strong acid or base in some cases [17,18,19,20]. On the other hand, the production of mesoporous metal based on the Ostwald ripening of nanoparticle aggregates in the absence of a template represents a novel method that promises high utilization efficiency of metal atoms. Moreover, when the porous nanoparticle aggregates are produced in a small dimension (for example, in nanoscale) with such a bottom-up technique, they can be loaded onto inexpensive catalyst carriers for applications with further enhanced utilization of precious metals [21,22]. However, it is challenging to control the partial aggregation of nanoclusters to avoid their dense packing. In this case, the number of active sites as a catalyst will decrease. Although some porous metal oxides have been prepared, the production of mesoporous metals by this method has rarely been developed [21,22,23,24,25,26].

In our previous work, we fabricated organic molecule-protected nanoparticle aggregate which was subjected to post-thermal ripening at 300 °C to form Pd-based mesoporous materials [27,28]. The porous materials have a large monomer size of hundreds of nanometers and a relatively small ligament size of 10–18 nm, and show high specific activity and excellent stability in electro-chemical methanol oxidation. Herein, to further enhance the utilization of precious metals by decreasing the whole size and ligament size of the porous material at the same time, the partial and controllable aggregation (self-assembly) of NPs was achieved based on a solvothermal reduction method, to form a coral-like structural Pd nano-catalyst (namely Pd NC). The fabrication was performed using a pair of weak reducing agents (i.e., oleyl amine and tetrabutylammonium bromide) at a relatively low temperature of 135 °C. The Pd NC with an average monomer (aggregate) size of 52 nm and average ligament size of 4.1 nm shows superior activity compared to conventional Pd NPs and the state-of-the-art Pt (under large potentials) for H_2_ evolution in alkaline water electrolysis.

## 2. Materials and Methods

### 2.1. Chemicals

Palladium (II) acetylacetonate [Pd(acac)_2_, 99%], tetrabutylammonium bromide, ReagentPlus (TBAB, 99%), trioctylphosphine oxide (TOPO, technical grade 90%), borane tert-butylamine complex (BTB, 97%), and oleylamine (OLA, technical grade 70%) were purchased from Sigma-Aldrich Co., Merck KGaA, Darmstadt, Germany. Activated carbon (Vulcan XC-72) was purchased from Cabot Performance Products (Tianjin, China) Co., Ltd. Acetic acid (>99.7%(T)) was purchased from KANTO CHEMICAL Co., Inc. (Tokyo, Japan). 5% Nafion(TM) Dispersion Solution DE521 CS type was purchased from FUJIFILM Wako Pure Chemical Co. (Osaka, Japan). Pt/C was purchased from Fuel Cell Earth (Livermore, CA, USA) (www.fuelcellearth.com, accessed on 24 April 2024) with a product name of Duralyst 10% Platinum on Vulcan XC-72.

### 2.2. Synthesis of Materials

A mixture of palladium acetylacetonate (0.2 mmol), tetrabutylammonium bromide (TBAB, 1.0 mmol), trioctylphosphine oxide (TOPO, 3 mmol), and oleylamine (6 mL) was deaerated using nitrogen gas for 3 min. After that, the reaction solution was heated to 135 °C and kept at this temperature for 2 h. After cooling down to room temperature, the mixture was diluted with ethanol (30 mL) and kept still for precipitation. The obtained solid material (Pd NC) was washed with ethanol (2 × 10 mL) and then dispersed on hexane (30 mL). In an Erlenmeyer flask, a suspension of carbon black (Vulcan XC-72) in hexane (140 mg in 50 mL) was subjected to ultrasonication for 20 min, into which the hexane solution of Pd NC was added. The mixture was subjected to ultrasonication for another 30 min. After evaporation of hexane, the material was treated by acetic acid (24 mL) at 65 °C for 6 h for surface cleaning. The obtained solid was washed three times with ethanol (30 mL, 15 mL, and 15 mL) and twice with hexane (15 mL and 15 mL) and then dried to form the Pd NC(N_2_) catalyst. When the deaeration procedure was avoided, Pd NC(air) was obtained.

Pd(BTB) was prepared based on the method for Pd NC(N_2_) with borane tert-butylamine complex (BTB) as the reducing reagent instead of TBAB. A mixture of palladium acetylacetonate (0.15 mmol), BTB (0.8 mmol), trioctylphosphine oxide (TOPO, 0.9 mmol), and oleylamine (3 mL) was deaerated using nitrogen gas for 3 min. After that, the reaction solution was heated to 130 °C and kept at this temperature for 2 h.

### 2.3. Characterizations of Materials

Transmission electron microscopy (TEM) and high-resolution TEM (HRTEM) were performed on a FE-TEM (JEOL JEM-2100F, Tokyo, Japan). The material was dispersed in ethanol and loaded onto a Cu microgrid for observation. The nitrogen adsorption isotherms were measured with a BELSORP-max gas/vapor adsorption analyzer, produced by MicrotracBEL Corp. (Osaka, Japan) Before nitrogen adsorption isotherm measurements, all the materials were pretreated for vacuum degassing at 150 °C for 5 h.

### 2.4. Electrochemical Experiments

All the electrochemical experiments were performed on an electrochemical analyzer, potentiostat/galvanostat (HZ-7000, MEIDEN HOKUTO Co., Tokyo, Japan). A conventional three-electrode cell was used, including a reference electrode of Ag/AgCl (filled with saturated KCl), a counter electrode of carbon rod, and a working electrode of the modified L-shaped glassy carbon electrode (with a surface area of 0.07 cm^2^, supplied by EC FRONTIER Co. Ltd., Tokyo, Japan). The potential value used in linear sweep voltammetry (LSV) and cyclic voltammogram (CV) profiles was changed from E(Ag/AgCl) to E(RHE), according to the formula E(RHE) = E(Ag/AgCl) + 0.2224 volts + 0.05916 × pH volts. For the electrocatalyst preparation, a mixture of catalyst (4.0 mg), Nafion (40 μL), distilled H_2_O (380 μL), and ethanol (380 μL) was ultrasonically dispersed for 30 min to form an ink. The L-shaped glassy carbon electrode was coated with the as-obtained catalyst ink and dried naturally at room temperature. Linear sweep voltammetry (LSV) experiments were performed without compensating *iR* drop in a N_2_-saturated 1.0 M KOH aqueous solution at 298 K.

The double-layer capacitance (C_dl_) measurements of different catalysts were carried out in N_2_-saturated 1.0 M KOH aqueous solution. The calculation of electrochemically active surface area (ECAS) is estimated from the C_dl_. The charging current density (*I*_c_) was measured from the CVs at different scan rates (5 mV/s~100 mV/s), as shown in Figure S3. The C_dl_ value can be calculated based on the equation *I*_c_ = νC_dl_, where *I*_c_ is current density and ν is the scan rate. *I*_c_ was determined by the equation *I*_c_ = (*I*_f_ − *I*_b_)/2, where *I*_f_ and *I*_b_ are the forward and backward currents, respectively.

## 3. Results and Discussion

**Figure 1:**
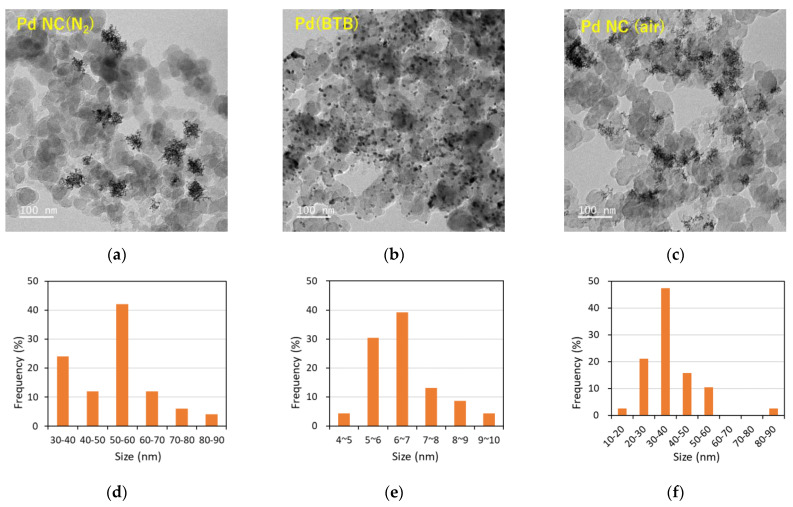
Transmission electron microscopy (TEM) images of Pd NC(N_2_), Pd(BTB), and Pd NC(air) supported on carbon (**a**–**c**), and their aggregate size (particle size in the case of Pd(BTB)) distribution information (**d**–**f**). Note that activated carbon XC-72 was used as the support here in all cases.

**Figure 2:**
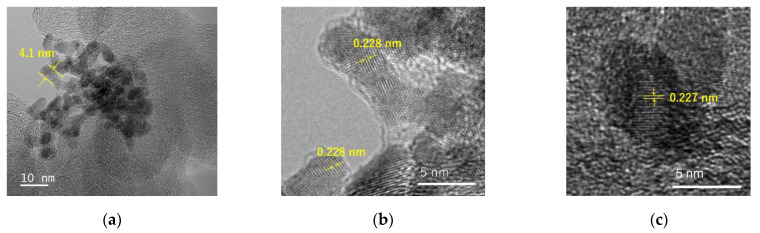
Magnified TEM image of Pd NC(N_2_) (**a**), high-resolution transmission electron microscopy (HRTEM) image of Pd NC(N_2_) (**b**), and HRTEM image of Pd(BTB) (**c**).

The Pd NC was fabricated with palladium(II) acetylacetonate (Pd(acac)_2_) as Pd precursor in a solvent of oleyl amine (OLA) which also acted as a capping reagent as well as a reducing reagent. Tetrabutylammonium bromide (TBAB) was used as a co-reductant (Table 1). The reaction mixture was bubbled by N_2_ gas and then heated at 135 °C for 2 h to form the mesoporous Pd NC(N_2_). The Pd NC(N_2_) was well distributed on carbon and had an avg. monomer size of 52 nm (Figure 1a,d). The magnified TEM image in Figure 2a shows that the Pd NC(N_2_) has a coral-like structure. On the other hand, the use of a strong reducing reagent, borane *tert*-butylamine complex (BTB), instead of TBAB resulted in the formation of monodisperse Pd NPs (named as Pd(BTB)), which are represented by black dots in Figure 1b, with an avg. size of 6.5 nm (Figure 1e).

**Scheme 1:**
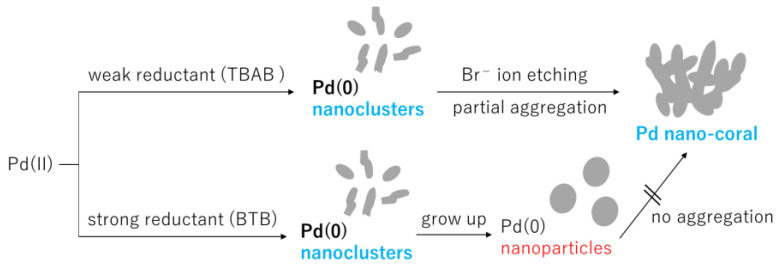
Schematic diagram of Pd(II) reduction for the formation of Pd NC and Pd NPs.

Interestingly, the mesoporous Pd NC can also be obtained under an air atmosphere, namely Pd NC(air). The avg. monomer dimensions of the Pd NC(air) were measured as 37 nm (Figure 1c,f). In a previous study by Sun et al., the solvothermal preparation of Pd nanostructures under a N_2_ or air atmosphere produced monodispersed Pd NPs or porous Pd particle aggregates, respectively [29]. It was suggested that oxygen existing in air resulted in the change of surface capping agent, which then induced the aggregation of Pd NPs. However, in our case, the porous Pd aggregate (Pd NC) can form either under a N_2_ or under an air atmosphere. It could be concluded that the formation mechanism of Pd NC is different from the previous one. As shown in Scheme 1, it was suggested that the intermediate Pd(0) nanoclusters could be partially etched by Br^−^ ion raised from TBAB and the etched parts became more unstable with high surface energies which went through partial aggregation to form the Pd NC [23,30]. When the fabrication was carried out without TBAB, it was observed that the color of the reaction mixture would not change into dark within the reaction time of 2 h, which might indicate that Pd(II) was hardly reduced. Moreover, when treating the resulting reaction mixture with ethanol, only a very small amount of precipitations was obtained, which also suggested that the yield of Pd nanomaterials was quite low. These results indicate that the addition of TBAB is necessary for both the reduction of Pd(II) to Pd(0) nanocluster and the aggregation of Pd nanocluster to form Pd NC. On the other hand, with the help of a strong reducing reactant, BTB, the Pd nanoclusters kept growing up quickly to form Pd NPs (Scheme 1). Without TBAB or Br^−^ ion, the Pd NPs would not aggregate any further.

Next, the morphologies of Pd NC(N_2_) with Pd NC(air) were compared. They possess a similar porous structure, as shown by the TEM images; however, the sizes of the aggregates were different based on the size distribution analysis (Figure 1d,f). For Pd NC(N_2_), 76% of the aggregates (nano-corals) are larger than 40 nm. In contrast, in the case of Pd NC(air), more than 71% of the aggregates are less than 40 nm. It indicates that the fusion of nanoclusters does not proceed well for Pd NC (air) in which the porous structure was less developed compared to Pd NC(N_2_). As the Br^−^ ion in TBAB could be oxidized by the O_2_ in air [31], the surface etching and the following aggregation of Pd nanoclusters would become inefficient with a decreased amount of Br^−^ ion under an air atmosphere. This could result in the less developed porous structure of Pd NC(air).

In Figure 2a, the mesoporous structure of Pd NC(N_2_) can be clearly seen and the ligament size of Pd NC(N_2_) is around 4.1 nm. The lattice space with an interplanar distance of around 0.228 nm is observed in the HRTEM image (Figure 2b), corresponding to the (111) reflections in face-centered cubic (*fcc*) Pd. Almost the same lattice distance could be determined in Pd(BTB), as shown in Figure 2c. For Pd NC (air), an avg. ligament size of 4.2 nm and a lattice distance of around 0.230 nm were determined by HRTEM (Figure S1).

To study the mesoporosity and surface area of the materials, a nitrogen gas physisorption experiment was carried out and the results were analyzed based on the Brunauer–Emmett–Teller (BET) and Barrett–Joyner–Halenda (BJH) methods. As shown in Table 2 and Figure S2, the mesopore volumes (*V_meso_*) of Pd NC(N_2_), Pd NC(air), and Pd(BTB) were calculated to be 25.28 cm^3^ g^−1^, 24.08 cm^3^ g^−1^, and 16.94 cm^3^ g^−1^, respectively. The pore volumes (*V_p_*) based on the BJH method also showed a similar trend. These results demonstrated the existence of mesoporosity in Pd NC(N_2_) and Pd NC(air), which partially contributed to the volume values of the two Pd NC. It is known that the mesoporosity of materials will increase their surface areas. In Table 2, the surface area of the Pd NC(N_2_) and Pd NC(air) was measured and found to be larger than that of Pd(BTB), based on either the BET (a*_s, BET_*) or the BJH (a*_p_*) method. In Figure S2c, the pore size distribution analysis was performed based on the BJH method. As the Pd(BTB) NPs do not have porosity, the pore size distribution data of Pd(BTB) could be assigned to the carbon black support (Vulcan XC-72). On the horizontal axis below 28 nm, the *dV_p_*/*dr_p_* values of Pd NC(N_2_) and Pd NC(air) are larger than the *dV_p_*/*dr_p_* value of Pd(BTB), indicating that the two Pd NC materials are probably rich in mesopores with a scale of less than 28 nm.

**Figure 3:**
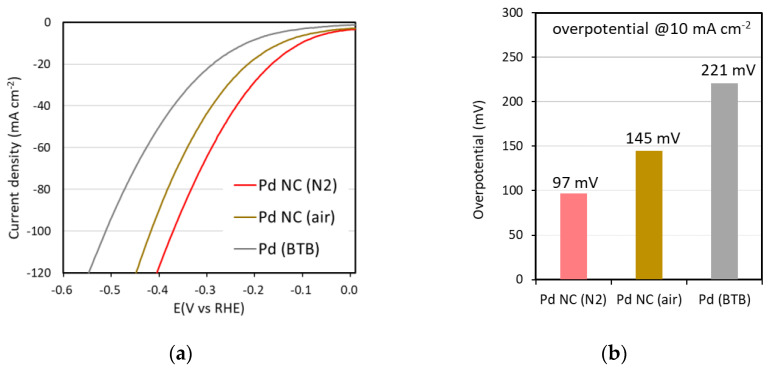

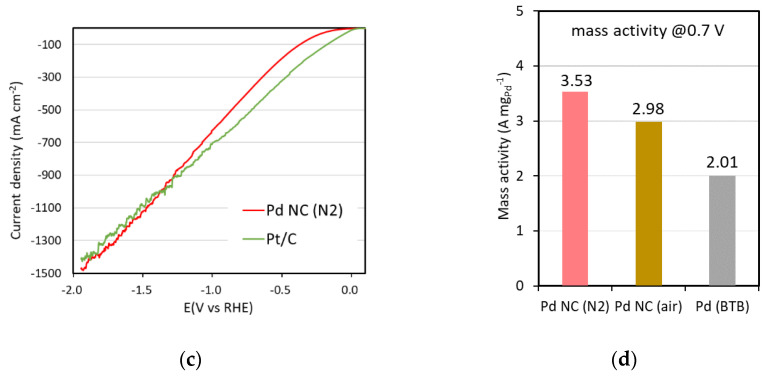
Electrochemical HER performance of Pd-based nanomaterials and commercial Pt/C. (**a**,**c**) LSV polarization curves; (**b**) summary of overpotentials at 10 mA cm^−2^; (**d**) calculated mass activity at −70 mV based on the mass of metallic Pd used for catalyst fabrication. All the experiments are recorded in N_2_-saturated 1.0 M KOH aqueous solution at around 298.15 K. The current density is equal to the current value normalized by the surface area of the working electrode.

The electrocatalysis of Pd NC(N_2_) and other related materials for HER was studied in a N_2_-satuated 1.0 M KOH aqueous solution. A three-electrode cell was used involving a modified L-shaped glassy carbon (LGC) as the working electrode, Ag/AgCl as the reference electrode, and a graphite rod as the counter electrode. Overpotential (η) is used to express the activity of an electrocatalyst in HER, and η_10_ represents the overpotential related to a current density of 10 mA cm^−2^. From linear sweep voltammetry (LSV) measurement (Figure 3a), Pd NC(N_2_) performs a η_10_ of only 97 mV with reference to the reversible hydrogen electrode (RHE, the same hereinafter). In contrast, the value is much higher with Pd NC(air) (145 mV) or Pd(BTB) (221 mV) as the catalyst (Figure 3b). Notably, Pd NC(N_2_) outperforms state-of-the-art Pt/C under high potentials (Figure 3c), showing a potential of Pd NC(N_2_) in practical alkaline-medium HER. To evaluate the utilization of precious metal, mass activities of different catalysts in the form of current density at −70 mV are calculated. As shown in Figure 3d, Pd NC(N_2_) affords the highest mass activity (3.53 A mg^−1^_Pd_), which is higher than that of Pd NC(air) (2.98 A mg^−1^_Pd_) and Pd(BTB) (2.01 mA mg^−1^_Pd_).

**Figure 4:**
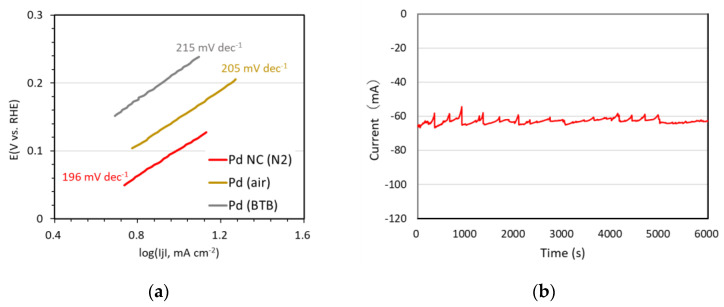

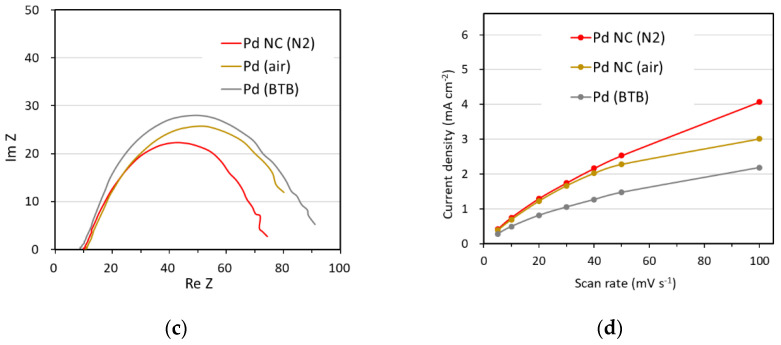
(**a**) Tafel curves of electrochemical HER performance; (**b**) CA test of Pd NC(N_2_) at a constant potential of 0.097 V vs. RHE; (**c**) Nyquist plots with a test frequency from 10^5^ Hz to 0.1 Hz; (**d**) electrochemical surface area calculation results based on C_dl_ in Figure S3. All the experiments were carried out in N_2_-saturated 1.0 M KOH aqueous solution.

Linear fitting of the LSV gives the corresponding Tafel curve as shown in Figure 4a, from which one can calculate the Tafel slopes. This value is useful for learning the HER kinetic and mechanism. The Tafel slopes of the three Pd-based catalysts were similar, ranging from 196 to 215 mV dec^−1^, indicating that the Volmer step is the rate-determined step for electrolysis of H_2_O into H_2_ [11]. The stability test of Pd NC(N_2_) was performed with chronoamperometry (CA) method at a constant potential of 0.097 volts (the η_10_ value of Pd NC(N_2_)). As illustrated in Figure 4b, the current density almost does not change during the 6000 s electrolysis process.

Electrochemical impedance spectra (EIS) and electrochemical double-layer capacitance (C_dl_) measurements determined the lowest charge transfer resistance (*R_ct_*) (Figure 4c) and the highest C_dl_ value (Figure 4d and Figure S3) of the Pd NC(N_2_) compared with those of other Pd-based catalysts. These results correspond to the exceptional HER activity of the Pd NC(N_2_). By comparison with Pd(BTB), the two porous catalysts Pd NC(N_2_) and Pd NC(air) show a lower *R_ct_* and a higher C_dl_. The calculated C_dl_ of Pd NC(N_2_) is 37.8 mF cm^−2^, which is nearly two times higher than that of Pd(BTB) (19.5 mF cm^−2^). These results indicate that the porous material has a good electrical conductivity and capacitance property, showing potential for practical electrolysis applications.

## 4. Conclusions

In summary, we have succeeded in the fabrication of a coral-like Pd nanomaterial with a mesoporous structure based on solvothermal reaction. The bottom-up synthetic procedure proceeds at mild temperature without any templates. It is suggested that the key points in the formation of the porous material is the use of TBAB to provide Br^−^ ion and the avoidance of a strong reducing reagent. As an electrocatalyst for the HER, the Pd NC(N_2_) showed high activity and stability in comparison with the traditional monodisperse Pd NPs. This work provides an atom-economic efficient method for the fabrication of mesoporous metallic nanomaterials without sacrificing any metal composites. The systemic research on the formation mechanism of the Pd NC is ongoing in our laboratory.

## Figures and Tables

**Table 1:** Pd nanomaterials with different morphologies fabricated under different conditions.

Material	Preparation Conditions ^1^	Material Morphology
Pd NC(N2)	standard conditions	nano-coral (porous aggregate)
−	no TBAB	Pd(II) hardly reduced
Pd(BTB)	BTB instead of TBAB	nanoparticle
Pd NC(air)	under air	nano-coral (porous aggregate)

^1^ Standard conditions: 0.1 mmol Pd(acac)_2_, 3 mL OLA, 0.5 mmol TBAB, 1.5 mmol TOPO, N_2_ atmosphere, 135 °C, 2 h.

**Table 2:** Textural properties of the three Pd-based materials analyzed by the BET and BJH methods based on the results of nitrogen adsorption isotherms *.

Sample	BET Method				BJH Method	
	*V_meso_*, cm^3^ (STP) g^−1^	a*_s_*, *_BET_*, m^2^ g^−1^	total pore volume (p/p_0_ = 0.99), cm^3^ g^−1^	average pore diameter, nm	V*_p_*, cm^3^ g^−1^	a*_p_*, m^2^ g^−1^
Pd NC(N2)	25.28	110.0	0.754	27.40	0.7332	82.67
Pd NC(air)	24.08	104.8	0.808	30.82	0.7898	86.60
Pd(BTB)	16.94	73.74	0.605	32.83	0.5978	71.04

* The nitrogen adsorption isotherms were carried out at 77 K. STP stands for standard temperature and pressure.

## Data Availability

The data presented in this study are available on request from the corresponding author, mingzhao@akita-nct.ac.jp. Data are contained within the article or the Supplementary Materials.

## Supplementary Materials

The following supporting information can be downloaded at https://www.mdpi.com/article/10.3390/nano14100876/s1: Figure S1: HRTEM image (a), ligament size distribution information based on Figure S1a (b), and a magnified HRTEM image marked with a lattice distance (c) of Pd NC(air); Figure S2: (a) Adsorption/desorption isotherm of the Pd-based materials. STP stands for standard temperature and pressure; (b) BET plots; (c) pore size distribution analysis based on the Barrett–Joyner–Halenda (BJH) method; Figure S3: Electrochemical double-layer capacitance (C_dl_) measurement of different electrocatalysts.
