# Supplementary material for: Solvothermal Fabrication of Mesoporous Pd Nano-Corals at Mild Temperature for Alkaline Hydrogen Evolution Reaction
Source: Nanomaterials (Basel). 2024 May 17;14(10):876. doi: 10.3390/nano14100876 (PMC11123806; doi:10.3390/nano14100876)
Supplement: Supplementary file 1 [file nanomaterials-14-00876-s001.zip › nanomaterials-3005306-supplementary.pdf]

Supplementary Information

Solvothermal Fabrication of Mesoporous Pd Nano-Corals at Mild Temperature for Alkaline Hydrogen Evolution Reaction

Ming Zhao [1,*], Koh-ichi Maruyama [1] and Satoshi Tanaka [2]

[1] Department of Materials and Biology, National Institute of Technology, Akita College, 1-1 Iijimabunkyocho, Akita 011-8511, Akita, Japan; maruko@akita-nct.ac.jp
[2] Department of Materials Science and Technology, Nagaoka University of Technology, 1603-1 Kamitomioka, Nagaoka 252-1123, Niigata, Japan; stanaka@vos.nagaokaut.ac.jp
* Correspondence: mingzhao@akita-nct.ac.jp

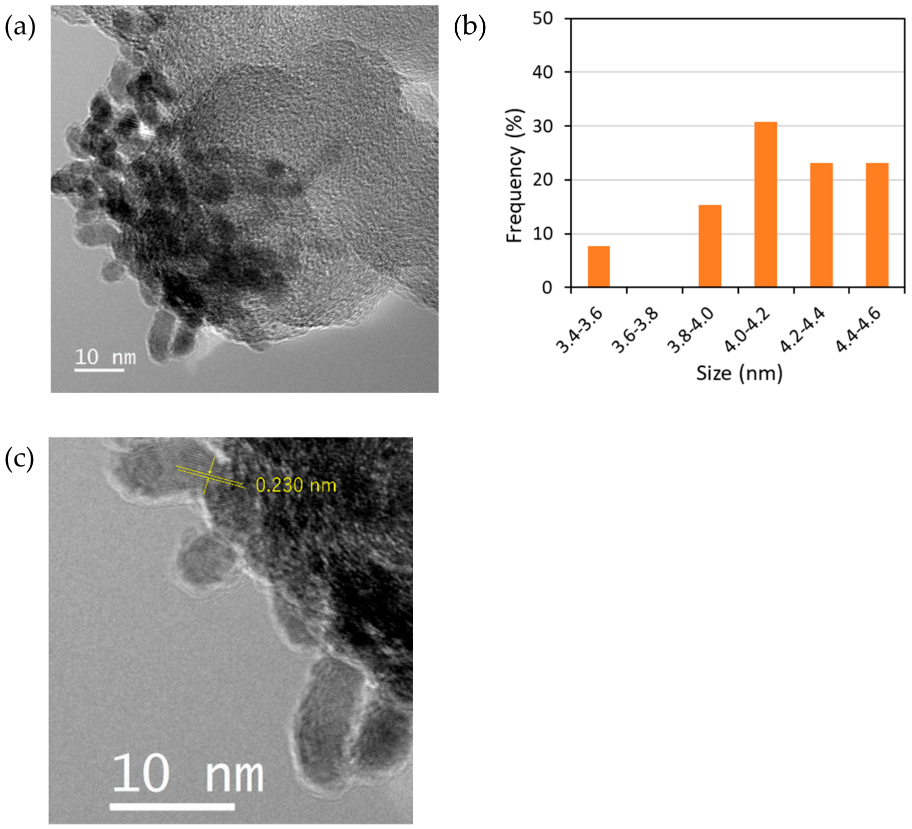

Figure S1. HRTEM image (a), ligament size distribution information based on Figure S1a (b), and a magnified HRTEM image marked with a lattice distance (c) of Pd NC(air).

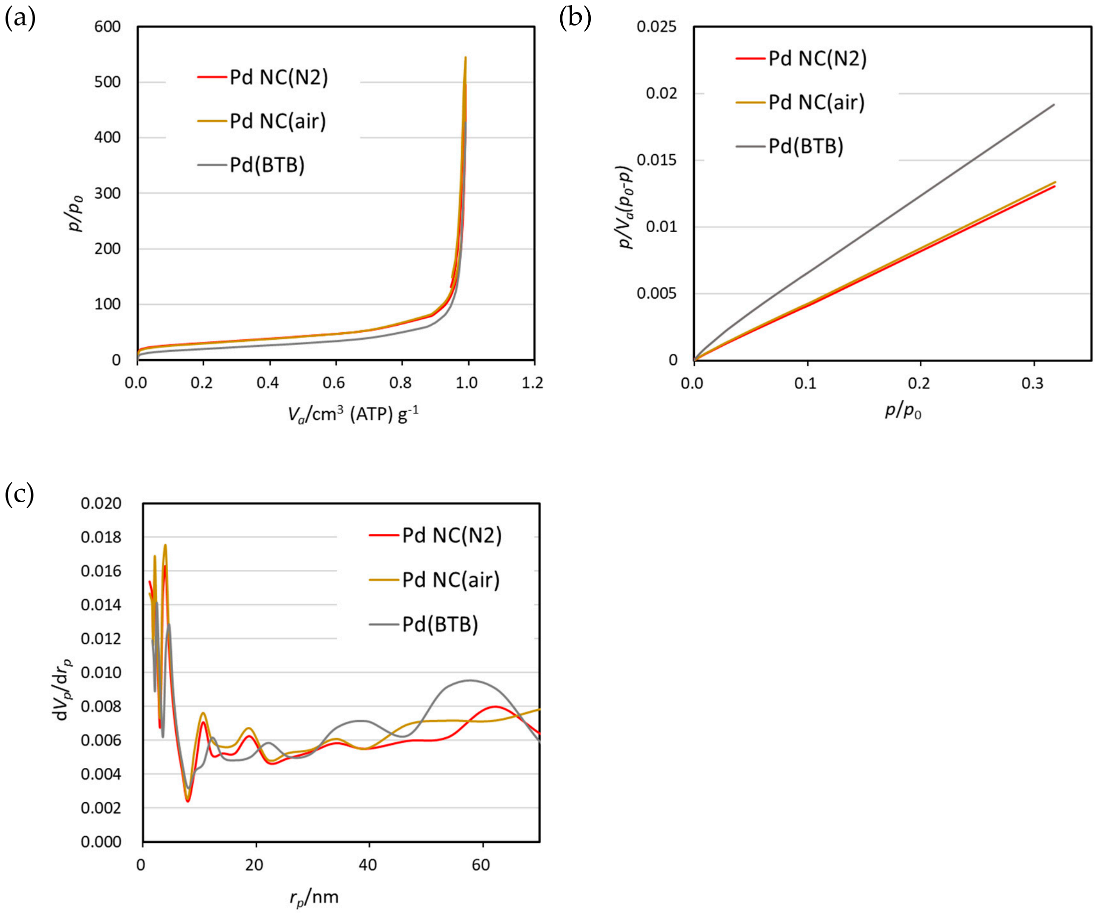

Figure S2. (a) Adsorption/desorption isotherm of the Pd-based materials. ATP stands for standard temperature and pressure; (b) BET plots; (c) Pore size distribution analysis based on the Barrett-Joyner-Halenda (BJH) method.

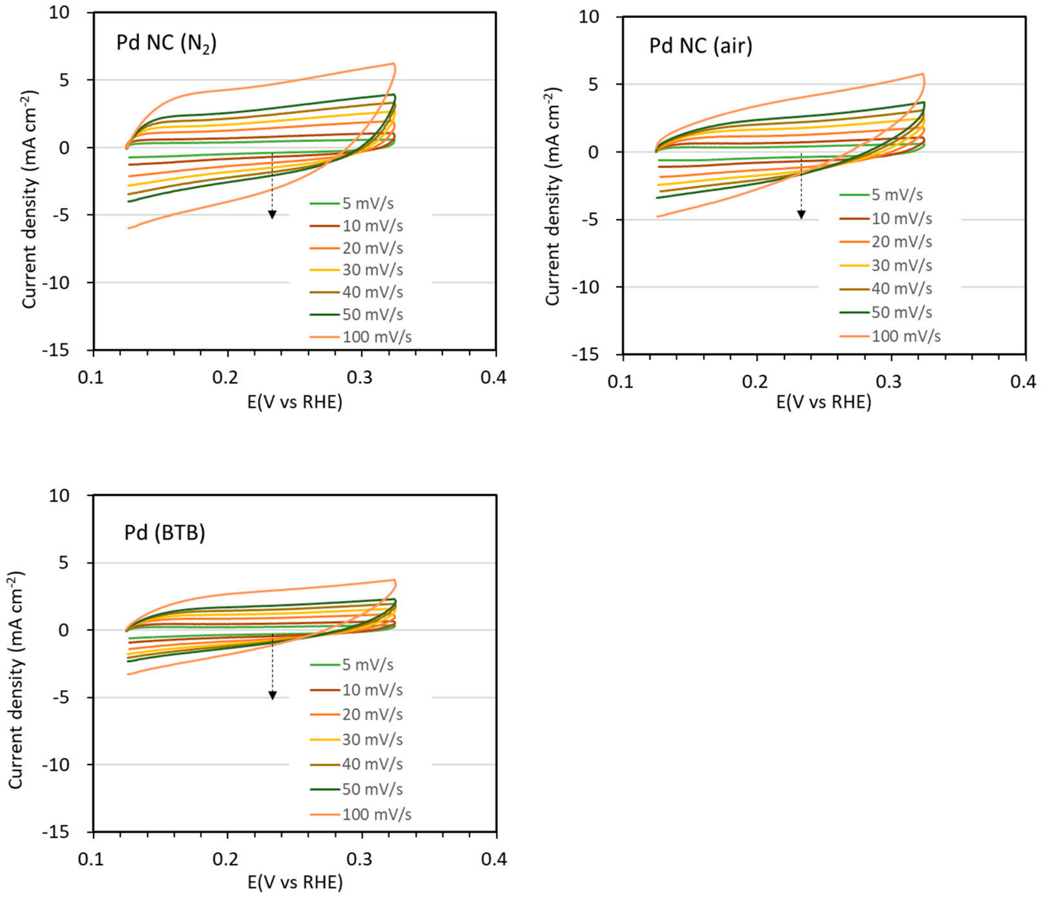

Figure S3. Electrochemical double-layer capacitance (C_{dl}) measurement of different electrocatalysts.
